# Successful Outcome after Treatment with Cidofovir, Vaccinia, and Extended Course of Tecovirimat in a Newly-Diagnosed HIV Patient with Severe Mpox: A Case Report

**DOI:** 10.3390/vaccines11030650

**Published:** 2023-03-14

**Authors:** Andres E. Martinez, Paola Frattaroli, Christine A. Vu, Lizy Paniagua, Joel Mintz, Andres Bravo-Gonzalez, Paola Zamudio, Astrid Barco, Aruna Rampersad, Paola Lichtenberger, Jose A. Gonzales-Zamora

**Affiliations:** 1 Division of Infectious Diseases, Department of Medicine, Jackson Memorial Hospital, Miami, FL 33136, USA; 2 Division of Infectious Diseases, Department of Medicine, Miller School of Medicine, University of Miami, Miami, FL 33146, USA; 3 Department of Pharmacy, Jackson Memorial Hospital, Miami, FL 33136, USA; 4 Department of Internal Medicine, University of Miami, Jackson Memorial Hospital, Miami, FL 33136, USA; 5 CES University, Medellin, Antioquia 050007, Colombia; 6 Universidad Anáhuac Querétaro, Querétaro 76246, Mexico; 7 Universidad de Especialidades Espíritu Santo, Guayas 092301, Ecuador; 8 Couva Hospital and Multi Training Facility, Couva 550214, Trinidad and Tobago; 9 Peruvian American Medical Society (PAMS), Albuquerque, NM 87111, USA

**Keywords:** immune reconstitution inflammatory syndrome, HIV, mpox, tecovirimat, cidofovir, vaccinia immune globulin

## Abstract

Purpose: To report a case of severe mpox in a newly diagnosed HIV patient concerning for Immune Reconstitution Inflammatory Syndrome (IRIS) and/or tecovirimat resistance and to describe the management approach in the setting of refractory disease. Case: 49-year-old man presented with 2 weeks of perianal lesions. He tested positive for mpox PCR in the emergency room and was discharged home with quarantine instructions. Three weeks later, the patient returned with disseminated firm, nodular lesions in the face, neck, scalp, mouth, chest, back, legs, arms, and rectum, with worsening pain and purulent drainage from the rectum. The patient reported being on 3 days of tecovirimat treatment, which was prescribed by the Florida department of health (DOH). During this admission, he was found to be HIV positive. A pelvic CT scan revealed a 2.5 cm perirectal abscess. Treatment with tecovirimat was continued for 14 days, along with an empiric course of antibiotics for treatment of possible superimposed bacterial infection upon discharge. He was seen in the outpatient clinic and initiated antiretroviral therapy (ART) with TAF/emtricitabine/bictegravir. Two weeks after starting ART, the patient was readmitted for worsening mpox rash and rectal pain. Urine PCR also returned positive for chlamydia, for which the patient was prescribed doxycycline. He was discharged on a second course of tecovirimat and antibiotic therapy. Ten days later, the patient was readmitted for the second time due to worsening symptoms and blockage of the nasal airway from progressing lesions. At this point, there were concerns for tecovirimat resistance, and after discussion with CDC, tecovirimat was reinitiated for the third time, with the addition of Cidofovir and Vaccinia, and showed an improvement in his symptoms. He received three doses of cidofovir and two doses of Vaccinia, and the patient was then discharged to complete 30 days of tecovirimat. Outpatient follow-up showed favorable outcomes and near resolution. Conclusion: We reported a challenging case of worsening mpox after Tecovirimat treatment in the setting of new HIV and ART initiation concerning IRIS vs. Tecovirimat resistance. Clinicians should consider the risk of IRIS and weigh the pros and cons of initiating or delaying ART. In patients not responding to first-line treatment with tecovirimat, resistance testing should be performed, and alternative options should be considered. Future research is needed to establish guidance on the role of Cidofovir and Vaccinia immune globulin and the continuation of tecovirimat for refractory mpox.

## 1. Introduction

Mpox is caused by the double-stranded DNA virus known as Human mpox Virus (MPXV), which belongs to the Poxviridae family [1]. Mpox was initially discovered in the 1950s, and the first human case was reported in 1970. Although sporadic reports of mpox have circulated worldwide since its discovery, a global outbreak in 2022 has led to a drastic rise in cases [2]. According to the CDC, there were 83,424 Mpox cases reported worldwide and 29,740 cases in the U.S. as of 1 January 2023, making the global rise in incidence a serious public health issue [3,4].

Mpox is a zoonosis that can spread through respiratory droplets, direct contact or fomites [1]. Historically, cases were transmitted from close contact with infected animals, with human-to-human transmission considered rare [5]. More recently, there has been increasing evidence of community spread, with a particularly notable incidence amongst men who have sex with men (MSM), which accounted for 98–99% of all new infections during the early stages of the epidemic [4,6,7,8]. Due to the high prevalence of HIV within the MSM population, there is also a connection between HIV and mpox, as approximately 30–40% of all patients diagnosed with mpox also had a diagnosis of HIV [6,9]. Immunosuppression from HIV, particularly in those undiagnosed or not taking antiretroviral therapy, may place them at risk of severe disease, which can lead to hospitalizations requiring ICU stays and death [10]. Others at risk of severe disease include pediatric patients, individuals with non-HIV-related immunodeficiencies, pregnant women, or those with medical conditions that impair skin integrity [11,12].

Symptomatic mpox generally progresses from fever to lymphadenopathy and cutaneous lesions, which usually resolve spontaneously without treatment. However, some patients can develop severe disease with the involvement of the oropharynx, face, and rectum [4]. Severe disease is defined by the CDC as the presence of multiple large confluent lesions, which can be hemorrhagic or necrotic, severe obstructive or necrotic lymphadenopathy, involvement of sensitive areas causing complications, including the genitals, anorectum, or oropharynx, superimposed bacterial infectious upon the lesions, or multiorgan system involvement [12]. For example, mpox patients may have concurrent ocular involvement, myocarditis, sepsis, acute kidney injury, pneumonitis or severe pharyngitis, amongst other complications. Historically the case-fatality rate of mpox was approximately 8.7%; however, much lower case-fatality rates of 0.03% have been reported during the recent epidemic [13].

No treatments have been approved for mpox; however, antiviral treatment, originally designed to treat smallpox, is recommended for patients with severe disease. Tecovirimat, an orthopoxvirus VP37 envelope-wrapping protein inhibitor, is generally considered a first-line treatment in the United States. For patients with progressive disease or life-threatening disease, vaccinia-immune globulin (VIVIG), cidofovir or brincidofovir can also be considered [14]. Of all mpox treatments, only cidofovir is approved for treating non-Poxviridae viral infections [15].

Here, we present a case of severe mpox in a newly diagnosed HIV-positive man that worsened despite multiple courses of tecovirimat and who was successfully treated with surgical intervention and multiple second-line antiviral therapies. We discuss the possibility of mpox-related immune reconstitution syndrome and resistance to Tecovirimat within the context of this patient’s case.

## 2. Case Report

A 49-year-old man, who self-identified as an MSM with unknown HIV status, presented to our emergency department (ED) with a 2-week history of painful itching perianal lesions. On a physical exam, he had two moderately sized pustular lesions in the perianal region along with small diffusely scattered pustular lesions over his face. Mpox PCR from pustular lesions was positive. Due to the patient’s mild disease, he was discharged home to isolate without treatment as the patient did not meet CDC criteria for treatment at this time. He declined HIV screening.

**Figure 1:**
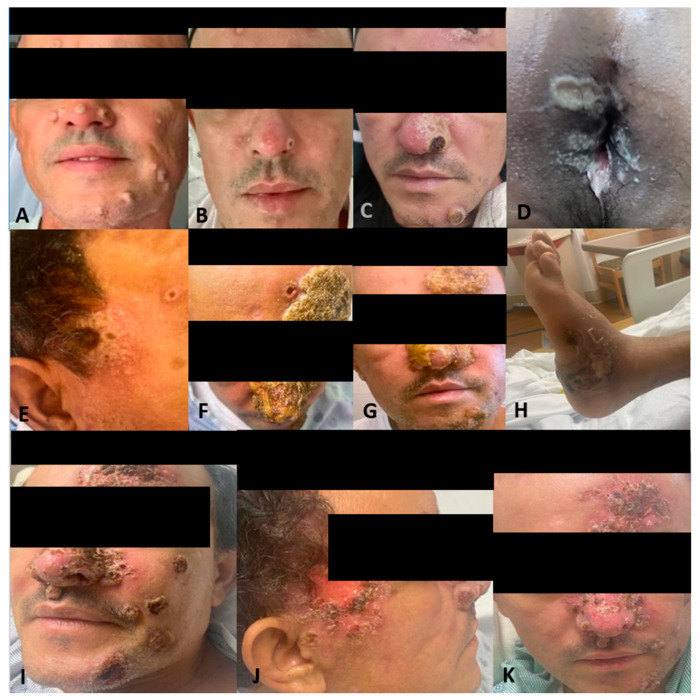
Patient presenting with monkeypox dermatological lesions: (**A**) Initial presentation without any previous treatment. (**B**) Improvement after treatment with tecovirimat. (**C**) Worsening of his monkeypox with hemorrhagic lesions with crusting in the center and whitish discoloration around them after 15 days of ART initiation. (**D**) White-yellow rounded punched-out erosions on the perianal region with elevated borders and surrounding purulent material. (**E**) Temporal lesion. (**F**,**G**) Worsening nasal lesions 42 days after ART initiation. (**H**) Abscess in the left ankle. (**I**) Face lesions after 1st Cidofovir dose. (**J**) Progression of the temporal lesion. (**K**) Lesions improving and crusted, close to discharge.

Two weeks later, the patient was seen at the Florida DOH and was started on tecovirimat 600 mg PO twice daily due to the progression and dissemination of his initial lesions. One week later, the patient returned to our ED, reporting burning pain with purulent discharge from his rectum. On examination, he had more than 25 skin lesions distributed on his face, scalp, mouth, chest, back, perineum and extremities. These lesions consisted of large confluent vesicles with central umbilication and ulceration (Figure 1A,B). The patient agreed to HIV screening, which was reactive. Further laboratory testing showed a CD4 count of 218 cells/mcL (CD4 percentage of 7.60%) and a viral load of 85,300 copies/mL. A pelvic CT scan was done, showing a 2.5 cm perirectal abscess. He was not tested for other sexually transmitted diseases during this admission. Antibiotic therapy with ceftriaxone and metronidazole was initiated for a presumptive superimposed bacterial rectal abscess. Treatment with tecovirimat continued as previously planned. After 4 days of therapy, the patient had an improvement in symptoms, including his skin lesions (Figure 1B). He was discharged on tecovirimat to complete 14 days of therapy along with a course of 7 days of amoxicillin-clavulanic acid for his abscess. Additionally, he was started on ART as an outpatient with TAF/emtricitabine/bictegravir.

**Figure 2:**
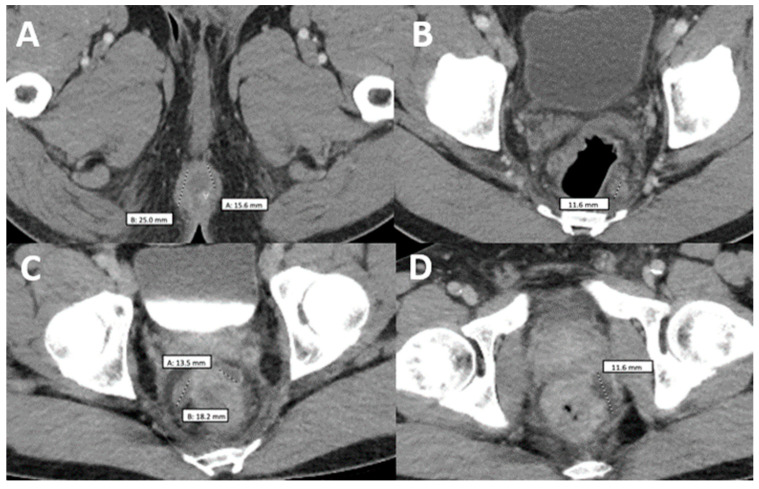
Comparison of two computerized tomographies of the pelvis with contrast. (**A**,**B**) were taken when the patient was already diagnosed with monkeypox but had not taken any medication and showed a rim-enhancing perirectal abscess measuring up to 2.5 cm in AP dimension (**A**) with a perirectal lymph node measuring up to 12 mm in short axis dimension (**B**–**D**) were taken when the patient came back after antiretroviral treatment with worsening of proctitis-related symptoms and showed three persistent vs. new rim-enhancing perirectal abscess.

Two weeks later and 6 weeks after the initial presentation, the patient returned to the ED after 5 days of the worsening of the existing Mpox lesions, bilateral periorbital edema, facial pain, and increasingly severe rectal pain. Of note, this was 15 days after starting ART and after completing the first course of tecovirimat for 14 days. Upon physical exam, he had numerous vesicles and pustules with central umbilication and necrotic crusting on his face (Figure 1C,E). Over his prior active Mpox lesions, he had hyperpigmentation and collarettes of scales, signs of excoriation with surrounding erythema. On his perirectal region, several rounded punched-out erosions with surrounding purulent discharge (Figure 1D). A second course of tecovirimat was initiated due to disease progression in the setting of a prior improvement with tecovirimat. Repeat abdominal/pelvic CT showed multiple perirectal abscesses indicating proctitis of the entire rectosigmoid segment. He underwent testing with urinary gonorrhea and chlamydia PCR, which was positive only for chlamydia. Due to presumptive lymphogranuloma venereum (LGV) proctitis, chlamydia urethritis, and perirectal abscess, the patient was started on oral doxycycline for 21 days as well as oral metronidazole and levofloxacin (Figure 2). At this time, surgical drainage was deferred. After 5 days of hospitalization, the patient’s symptoms improved, and he was discharged on a second 14-day course of tecovirimat, along with his other antimicrobial regimens and prophylaxis.

With one day remaining in his second Tecovirimat course and 8 weeks after initial presentation, the patient was readmitted to the hospital for worsening mpox symptoms and persistent severe rectal pain, unabated by antibiotic treatment for LGV proctitis and bacterial abscess prescribed on his most recent hospitalization. He reported difficulty breathing due to partial blockage of the nasal airway by Mpox lesions on his nose (Figure 1F,G). A third course of Tecovirimat was started, given his clinical presentation. The repeat CD4 count was 257.28 cells/mcL (8%), and the HIV viral load of 203 copies/mL.

During this hospitalization, the perineal abscess was drained, as well as a new abscess on his left ankle (Figure 1H). Furthermore, despite being on a third course of tecovirimat, new lesions were still appearing. Given persistent mpox symptoms, the CDC Mpox Clinical Consult team was contacted for guidance. The decision was made to administer Cidofovir 5 mg/kg weekly with Probenecid and Vaccinia immune globulin 9000 U/kg. The patient received a total of three weekly doses of Cidofovir and two doses of Vaccinia immune globulin. Additionally, two wound swabs taken from a facial lesion and the gluteal area were performed and sent to the CDC for non-CLIA-certified tecovirimat resistance testing. After the 1st dose of Cidofovir and Vaccinia, skin lesions started to improve (Figure 1I–K), with areas over the face crusting with a remarkable reduction in the number of lesions covering the nose and temporal area. The patient still reported rectal pain but with overall improvement. Given signs of clinical improvement, pending resistance testing, and continued discussion with the CDC, the patient was ultimately discharged on Tecovirimat 600 mg PO BID for 30 days. Upon the patient’s first and second month outpatient follow-up, he has achieved near complete resolution of his lesions.

## 3. Discussion

The coinfection and superinfection between HIV and mpox have been well established, and its incidence is estimated to be around 40% in multicenter studies [14]. Both diseases are considered sexually transmitted infections (STI) and have significantly impacted the MSM population [14,15]. Both viruses are considered to be pro-inflammatory, which increases the probability of transmission of each other. On one side, mpox induces an inflammatory response that recruits CD4+ lymphocytes making it a local target for the HIV virus to invade the skin. On the other hand, the immunodeficiency created by HIV hinders the viral clearance from the mucosa, which increases the chances of mpox infection [16].

Mpox can infect HIV-positive individuals independent of CD4+ count [17]. Classic mpox presents with fever, lymphadenopathy, and generalized skin rash, which is characterized as firm, deep-seated, well-circumscribed, painful, and umbilicated lesions, which can include palms and soles, but in HIV patients, this infection tends to present with a lack of fever and predominant anogenital lesions [18,19]. This new 2022 outbreak of mpox has manifested more extensively in MSM patients, occurring more often in people living with HIV. Our patient presented with lesions in the perianal area, face, scalp, mouth, chest, back, legs, and arms. Furthermore, patients with mpox and HIV coinfection have a more prolonged illness, larger lesions and higher rates of both secondary bacterial skin infections and genital ulcers [20]. Closer attention should be paid to HIV-coinfected patients, even those with undetectable viral load and normal CD4+ lymphocyte count [21].

Our patient initially came with classical mpox skin lesions and started improving after the first course of tecovirimat treatment (Figure 1B). Then, he was diagnosed with HIV and had borderline immunosuppression (CD4 count of 218 cells/mcL and a percentage of 7.60%) with subsequent ART initiation. However, the skin lesions took a paradoxical turn after 4 weeks and apparently, the only precipitant factor identified after extensive workup was ART. IRIS was previously described as a syndrome that affects patients with a low baseline CD4 count, below 100 cells/mcL for most infections except for tuberculosis which can occur regardless of the immune status [22,23]. Nevertheless, further studies have shown that there are other factors that can induce the response in non-lymphopenic patients, like the dysregulated antigen recognition when the repopulation happens, explained by the higher frequencies of effector memory, PD-1+, HLA-DR+, and Ki67+ CD4+ T cells/mcL [24]. For example, in a case report of a patient with HIV and IRIS-related *Pneumocystis jiroveci* pneumonia, the CD4 count was 370 cells/mcL [25]. Also, in the TB-HAART clinical trial, a cohort of more than 1500 patients diagnosed with tuberculosis and with a CD4+ count greater than 220 cells/mcL was evaluated, and a 10% incidence of tuberculosis-associated IRIS was found [26]. Furthermore, since the introduction of Integrase strand transfer inhibitors (InSTIs), the viral load can be more rapidly suppressed than the cases treated with other regimens accelerating the lymphocyte repopulation, and this can also explain why the syndrome happened so early in the disease course (15 days from ART initiation to first IRIS symptoms) [27]; however, a recent meta-analysis found that there is no overall increased incidence of IRIS when using InSTIs compared to alternative regimens [28]. Although there is no way how to confirm that the worsening of lesions on this patient corresponds to IRIS, it is highly suggestive that the initiation of ART may play a role in the worsening of pre-existent lesions and, thus, suggest paradoxical mpox IRIS in this population.

Simon-Gozalbo et al. described an unusual yet similar case of a hemorrhagic mpox in a patient with HIV (CD4+ T-cell count of 265 cells/mL) who had recently initiated ART. However, they mention that IRIS was low on the differential because the patient did not have AIDS or a CD4 count below 200 cells/mcL and because there was no evidence suggesting a pre-existing mpox infection that was being exacerbated by the ART initiation, so it could have been a completely new infection as well [29]. In contrast with our case, this patient did not develop chlamydia proctitis and did not have a clinically significant and confirmed infection of mpox that was later exacerbated, most likely by IRIS [30,31]. Interestingly, this patient had a nucleic acid amplification anal swab test positive for chlamydia and was treated with doxycycline; however, he never presented proctitis like in our case. In terms of ART, it was continued through the suspected IRIS, given that the treatment suspension has been associated with rebound viremia and worsening of illness [32,33,34]. Empiric treatment with steroids to reduce the inflammatory response was considered but not done. There is no data favoring or against this indication since this is the first case reported; however, there are certain indications for steroid treatment, such as IRIS secondary to Cryptococcus meningitis, *M. tuberculosis* meningitis or JC virus Progressive multifocal leukoencephalopathy.

Our patient’s clinical presentation was deemed refractory to tecovirimat treatment after failing to improve after two courses of tecovirimat therapy. While the optimal duration of tecovirimat treatment for mpox is unknown, initial courses of 14 days have been recommended by the CDC based on data extrapolated from human smallpox studies caused by the variola virus [35]. More recently, real-life experience of tecovirimat for mpox has been published and suggests that longer durations may be warranted for select cases, particularly in patients who are immunocompromised. An MMWR report by the CDC described two patients with AIDS that required prolonged treatment with tecovirimat: one patient required more than 4 weeks of oral and intravenous tecovirimat, and the other patient still had progressive necrotic lesions after 7 weeks of oral tecovirimat therapy [36]. In terms of our patient, there were signs of new lesions even after starting the 5th week of tecovirimat treatment. While there was discussion on switching to an intravenous formulation of tecovirimat to improve bioavailability, we opted not to pursue it due to the patient reliably taking the medication with high-fat meals and no identifiable absorption issues. Due to the lack of response to oral tecovirimat, we had a high suspicion of drug resistance.

To date, there have been limited reports of tecovirimat resistance. Our patient had risk factors for developing resistance due to receiving intermittent and prolonged therapy. It has been described that tecovirimat has a low barrier to developing resistance, particularly in situations of overprescribing. In vitro studies have shown that resistance can be caused by alterations in single base changes within the virus genome that encode the p37 protein, which is the target site for tecovirimat [37]. To date, there have been three cases reported with tecovirimat resistance. One patient with acute myeloid leukemia developed Orthopoxvirus resistance after 3 weeks of oral tecovirimat, and two patients with AIDS who developed mpox resistance, both of whom were severely immunocompromised and received prolonged treatment beyond 14 days [37,38]. For our patient, the isolate was sent out to the CDC for sequencing and resistance testing, but unfortunately, these results could not be released due to laboratory regulations, with the test not yet being CLIA-certified.

Due to our patient’s lack of clinical response and concern for tecovirimat resistance versus IRIS, we explored other treatment modalities. After discussion with the CDC Clinical Task Force, we decided to initiate 2 doses of Vaccinia immune globulin 9000 U/kg and cidofovir 5 mg/kg weekly as an adjunct therapy. Data to support cidofovir comes from animal studies and a case series from Italy during a time when tecovirimat was not yet available [39,40,41]. Four hospitalized men had severe mpox and received cidofovir; all patients experienced resolution of lesions, fever, and lymphadenopathy after just one dose. One of these patients even had greater than 10 vesicular lesions on the conjunctiva and had complete resolution after cidofovir treatment. Another case was reported in the United Kingdom, where a patient with poorly controlled HIV and Ludwig’s angina was treated with tecovirimat and subsequently with topical and intravenous cidofovir, after which he experienced significant improvement [42]. Fabrizio et al. also described an HIV patient with severe mpox treated only with cidofovir. Of note, this patient experienced resolution of his symptoms after two doses of cidofovir [43]. A more recent case series was reported by Mondi et al., who documented their clinical experience with 19 mpox patients, of whom four were treated with cidofovir and had successful outcomes [44]. Likewise, our patient developed improvement in his lesions soon after the first dose of Cidofovir.

Vaccinia immune globulin was also provided by the CDC around the same time. A case report was recently published about a man with AIDS who did not respond to tecovirimat but instead showed clinical improvement after Vaccinia immune globulin was added for the treatment of refractory mpox [45]. It is unclear whether our patient’s clinical response was due to Cidofovir or Vaccinia treatment, or both since they were administered only 2 days apart. Based on this experience, it would be important to consider IRIS in the setting of mpox and to evaluate the benefits versus risks of delaying ART initiation in this setting. Evidence regarding the effectiveness of tecovirimat in the treatment of severe mpox in the immune-compromised host is needed, as well as the possible role of Cidofovir and Vaccinia immune globulin for the treatment of refractory severe mpox cases.

## 4. Conclusions

We present the first case of refractory mpox in a newly diagnosed HIV patient with possible IRIS and tecovirimat resistance. Our case highlights the complexities in the management of mpox in an immunocompromised host who does not respond to first-line therapy. Further research is needed on the role of tecovirimat in these situations to determine whether treatment should be extended or discontinued, along with selecting the appropriate adjunct therapies. Providers should have a low threshold to pursue alternative treatment options such as cidofovir and vaccinia immune globulin, especially as there is now real-world experience demonstrating its benefits. It is also important to acknowledge the potential role of mpox-related IRIS in patients who initially worsen after starting tecovirimat, especially if concomitantly started on antiretrovirals. Finally, we believe that the timing of mpox treatment and initiation of ART should be carefully considered in the management of patients with HIV.

## Data Availability

The data that support the findings of this study, after adequate anonymization that protects the patient’s privacy, will be available on request from the corresponding authors. The data are not publicly available due to them containing information that could compromise research participant privacy/consent.
